# SpiKon-E: Hybrid Soft Artificial Muscle Control Using Hardware Spiking Neural Network

**DOI:** 10.3390/biomimetics10100697

**Published:** 2025-10-15

**Authors:** Florian-Alexandru Brașoveanu, Mircea Hulea, Adrian Burlacu

**Affiliations:** 1 Department of Automatic Control and Applied Informatics, Faculty of Automatic Control and Computer Engineering, “Gheorghe Asachi” Technical University of Iasi, Str. Dimitrie Mangeron, 700050 Iași, Romania; florian-alexandru.brasoveanu@academic.tuiasi.ro; 2 Department of Computer Science, Faculty of Automatic Control and Computer Engineering, “Gheorghe Asachi” Technical University of Iasi, Str. Dimitrie Mangeron, 700050 Iași, Romania; mircea.hulea@academic.tuiasi.ro

**Keywords:** soft robotics, spiking neural network, system control

## Abstract

Artificial muscles play a key role in the future of humanoid robotics and medical devices, with research on wire-driven joints leading the field. While electric servo motors were once at the forefront, the focus has shifted toward materials that react to changes in the environment (smart materials), including pneumatic silicone actuators and temperature-reactive metallic alloys, aiming to replicate human muscle actuation for improved performance. Initially designed for rigid actuators, control strategies were adapted to address the unique dynamics of artificial muscles. Although current controllers offer satisfactory performance, further optimization is necessary to mimic natural muscle control more rigorously. This study details the design and implementation of a novel system that mimics biological muscle. This system is designed to replicate the full range of motion and control functionalities, which can be utilized in various applications. This research has three significant contributions in the field of sustainable soft robotics. First, a novel shape memory alloy-based linear actuator is introduced, which achieves significantly higher displacements compared to traditional SMA wire-driven systems through a guiding mechanism. Second, this linear actuator is integrated into a hybrid soft actuation structure, which features a silicone PneuNet as the end effector and a force sensor for real-time pressure feedback. Lastly, a hardware Spiking Neural Network (HW-SNN) is utilized to control the exhibited force at the actuator’s endpoint. Experimental results showed that the displacement with the control system is significantly higher than that of the traditional control-based shape memory alloy systems. The system evaluation demonstrates good performance, thus advancing actuation and control in humanoid robotics.

## 1. Introduction

In the rapidly evolving field of robotics, soft robotics has emerged as a promising subfield that addresses the limitations of rigid robots. While rigid robots have benefited from decades of development, which offer robust solutions for numerous applications, they face persistent challenges in terms of flexibility of motion, resistance to external contact, and safety concerns in open environments.

Soft robotics offers an innovative solution, emphasizing intrinsic safety, adaptability, and novel motion capabilities [1]. These features make soft robots particularly suited for collaborative environments, including the medical field, where their flexibility and safety can enhance performance and usability.

Recent advances in soft actuators have focused on two primary areas: actuation performance and control methods [2]. Actuation performance is evaluated based on several key factors, including force, repeatability, stability, energy consumption, and actuator volume. On the other hand, control methods have evolved from classical approaches to advanced techniques, such as deep learning and neural networks, enabling precise and adaptive functionalities. These developments underline the potential of soft actuators to serve as foundational components in cutting-edge robotic systems, particularly in medical applications.

Medical robotics often employs flexible structures to minimize contact damage and improve safety, distinguishing them from rigid robots used in heavy-duty applications. Soft actuators play a pivotal role in this domain, offering innovative motion dynamics such as directional bending [3,4] and volume extensions [5,6]. These capabilities make them suitable as standalone robots [7] or as end-effectors [8,9] in complex systems.

One significant area of study in soft robotics is the use of shape memory alloys (SMAs). These alloys are lightweight and highly resistant, making them ideal for applications that require motion generation. However, challenges such as the size-to-displacement ratio and actuation speed remain, requiring advancements in design and modeling [10]. Applications integrating SMA wires have key aspects to be considered, as presented in [11].

SMAs are a widely recognized solution among intelligent materials used in soft robotics. They are prized for their lightweight properties, strong actuation forces, and versatility, enabling their integration into a broad range of applications. Their adaptability has made them a core component in innovative devices across various fields, including civil infrastructure [12], aeronautics, and medicine [13]. SMAs enhance resistance to severe environmental conditions in civil engineering, as noted in [14]. In aeronautical systems, the torque capabilities and lightweight properties of SMAs make them ideal for wing-bending mechanisms, offering flexibility and vibration resistance. Automotive and medical systems further benefit from SMAs, as described in [15], highlighting their adaptability and strong force-to-dimension ratio. However, challenges such as hysteresis behavior and thermal-based response times remain significant drawbacks, as outlined in [16]. Mechanical design innovations and environmental management strategies are necessary to mitigate these issues and enhance performance.

Efforts to optimize SMA-based actuators have included parallel wire configurations to enhance force output [17] and customized designs to balance performance and control [18]. Linear SMA actuators are particularly relevant in rehabilitation and prosthetics, where adaptability to constrained spaces and precise force control are critical [19]. Furthermore, the SMA wires have been tested and integrated into various biomimetic structures focused on angular control [20].

Simulations of soft actuators face challenges due to the complex behavior of soft materials and systems. As discussed in [21,22], achieving high simulation accuracy often depends on the use of detailed models and the manipulation of internal parameters. While generalized testing methods remain elusive, specific strategies have shown remarkable results. For instance, modeling approaches that consider modular strokes [23] and broader physical perspectives [24] have provided significant insights. Moreover, simulation environments like SoRoSim have enabled researchers to test and validate these systems effectively [25]. Nevertheless, the computational power required for highly detailed models, as noted in [26], can sometimes exceed practical requirements for specific simulations. In [27], the numerical simulation of a hybrid actuator system as an integration of two mathematical models is discussed, along with the effect, acting as a strong foundation for a physical implementation.

Spiking neural networks are the third generation of NNs that demonstrate the highest performance in modeling the behavior and learning abilities of the brain. The SNN benefits from the increased computational power and accuracy compared with other types of NNs, including the traditional ANNs [28]. This is because an SNN operates based on precise timing spikes, which make it sensitive to time-varying functions and random occurrences of events [29].

The context provided reveals the primary challenges in soft robotics today, namely limitations in actuation performance and the need for advanced control strategies.

To address these challenges, this study aims to contribute to the state of the art in soft robotics by developing a novel complex system that mimics the functions of natural musculoskeletal structures. It contains a novel SMA wire-based linear motor integrated into an advanced hybrid soft actuator, accompanied by a hardware neural network architecture designed for precise force control in biomedical systems.

This design overcomes prevailing limitations by compactly embedding extended SMA wires, thereby enhancing actuation efficiency and system performance by effectively doubling the achievable displacement while preserving a lightweight body. The hybrid actuation system combines the benefits of SMA wires and PneuNet elasticity, resulting in enhanced dynamics. The force control employs a force-sensing resistor providing feedback to the analogic HW-SNN architecture that controls the pressure generated in auctioning. Overall, it delivers a natural finger movement, emphasizing the force exhibited at the tip of the finger.

Section 2 provides an extensive overview of the solution, followed by a Section 3 that presents the core materials and functionalities used. The following Section 4 presents the development of the system and the integration of each component. Section 5 presents the analysis of actuator dynamics and complete system performance, demonstrating the advancements of the solutions. The final Section 6 presents conclusions for the approach and outlines future perspectives.

## 2. Problem Formulation

**Figure 1:**
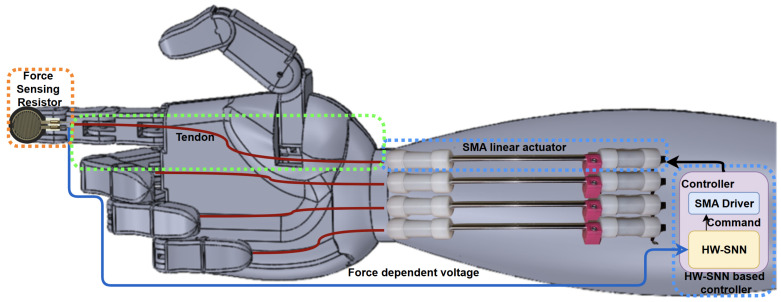
The structure of an anthropomorphic robotic hand that can use the proposed control system.

The contributions claimed in this paper have a high potential for applicability. Figure 1 displays a scaled integration of multiple such systems in an operable fashion within a hand prosthesis [30]. The primary characteristic of the complete system is its ability to be operated and integrated without requiring preparatory operations, such as controller parametrization or calibration. Although the silicone PneuNet is omitted from the figure for clarity, it replaces the mechanical finger, embedding the force sensor within its volume.

The linear actuator present in the scheme represents a novel mechanical design that overcomes the SMA linear displacement limitation by using a longer wire to achieve greater deformations. By doubling the basic linear displacement of the SMA wire while maintaining a relatively lightweight structure, this actuator could represent a breakthrough in future prosthetic design.

The actuator is therefore part of a hybrid actuation system, which also contains one of the most popular soft robotic structures: a pneumatic network. As the hybrid configuration benefits from the qualities of both components, it can compensate for the reciprocal downsides.

To demonstrate the applicability of the actuator, a suitable control structure is necessary to achieve behaviors that closely resemble those of biological systems. The HW-SNN architecture employed in the control of a hybrid soft system represents a pioneering approach, bridging the gap between technological medical replacement devices and the functional capabilities of biological muscle.

Using a spiking neural network allows the control structure to be placed closer to the motion logic of spinal neurons, offering an accurate and authentic representation of neuromuscular control. The HW-SNN can be easily embedded in the prosthesis’s inner layers, and the actuators actuate each finger individually. With enhanced performance and ease of integration, the system represents a foundational advancement toward fully functional prosthetics, thereby advancing the field of soft robotics and its impact on human-centered technology.

## 3. Materials and Methods

The developments described further utilize modules and elements from various domains, including digital and analog electronics, materials science, embedded systems, and control theory. The key elements that require detailed understanding are the SMA as a material in wire form and HW-SNN primary concepts and usage. The following specific topics are detailed below, with an emphasis on their core features.

### 3.1. Shape Memory Alloy Actuation

Motion dynamics in soft actuation are often governed by the molecular aggregation of various chemical components, which can be influenced by external or internal factors. On a macro-scale, soft actuations typically depend on simple physical phenomena generated by materials or structural designs. While materials alone can create basic displacements, achieving guided and purposeful movement requires tailored physical arrangements or encapsulating mechanisms.

SMAs represent a significant subgroup of intelligent materials within the field of soft robotics. They are characterized by their intrinsic reactions to environmental inputs, which are based on their internal atomic structure. Notably, their size, speed, and exceptional electrical conduction properties make them the most suitable material for the current research.

Developing soft actuators with SMAs heavily depends on the alloy’s composition and its initial form. Alloy composition determines mechanical limitations, energy requirements, and operational efficiency. For instance, ref. [31] offers a detailed analysis of SMA alloys, focusing on structural composition, energy consumption, and damping capabilities. Additionally, a theoretical framework for energy behavior at the microstructural level is explored in [32], providing valuable parametric insights into the functionality of SMA.

SMAs can be activated through current-driven methods, which rely on temperature changes to induce shape transformations. In environmental-driven approaches, SMAs are placed in controlled environments where temperature modifications trigger the desired actuation. An example in [33] describes a linear actuator with parallel SMA wires in a chamber traversed by fluid and a heating element to regulate temperature. Alternatively, current-driven methods utilize electrical currents to generate thermal energy directly within the alloy, allowing for precise control over motion.

### 3.2. Hardware Spiking Neural Network

The functional and structural units of SNN are the spiking neurons that introduce timing and adaptability to information processing, making the temporal occurrence of events an essential parameter in such networks. This type of neuron models the natural mechanisms that govern the operation of the biological neurons, such as temporal integration of incoming stimuli, activation threshold, and refractory period. In addition, SNs mimic the natural mechanisms that determine synaptic plasticity, including long-term potentiation and depression, which increase or reduce synaptic weights [34]. Depending on the type of generated stimuli, the neurons can be excitatory or inhibitory, and the amplitude of the postsynaptic response depends on the weights.

Typically, spiking neurons model the key characteristics of neural cells, including spiking behavior, the integration of incoming stimuli, the detection of the activation threshold, the refractory period, and the generation of excitatory or inhibitory stimuli. In addition, the artificial model of neurons incorporates properties related to synaptic plasticity, such as long-term potentiation and depression, which alter the synaptic weights. Typically, the SNs communicate by discrete stimuli that determine the postsynaptic response, whose intensity depends on the synaptic weights [34].

The SNNs are suitable for hardware implementation [35,36] because they are based on the parallel operation of a significant number of high-complexity spiking neurons with integrate-and-fire characteristics. The NNs based on such neurons benefit from low power consumption, excellent signal-to-noise ratio, and fast response due to the parallel operation of the neurons, making them a good option for decoding the data generated by event-based sensors.

Significant attention has been given to the complex adaptability mechanisms [37,38] of SNNs. Several learning methods [39], including the gradient descent [40], are successfully implemented in deep SNNs, while another method called e-prop was introduced in [41]. The latest method, which is based on the backpropagation of errors, may explain the learning mechanism in recurrent spiking neural networks. However, the most rigorous models of neurons implement the plasticity rules of the biological synapses, which include the spike-timing-dependent plasticity (STDP) [42,43].

The primary challenge for hardware SNNs is the storage and real-time adjustment of the synaptic weights. To fulfill these requests, digital methods such as the random access memory (RAM) buffers and phase change memory (PCM) [44] are implemented. The weights can also be stored in analog hardware using floating gate transistors (FGT) [45] or memristors [46,47], which represent a recently implemented circuit element.

Recent research shows that the memristor-based technology can be used to implement devices that mimic the adaptability [48] of the biological synapses, as well as the generation of pulses with different frequencies [49]. Another advanced material developed to implement artificial synapses is the multilayer hexagonal boron nitride (h-BN) [50]. However, a cheap and straightforward method for storing synaptic weights is represented by capacitors, which allow for real-time adjustment of the weights using elementary electronic circuits. However, the main disadvantage of this method is the leakage currents, which additional pulse generators can compensate for when weight refreshing is needed.

## 4. Development

This research examines actuation and control separately, with each validated through specific testing and numerical analysis. While the first aims to demonstrate the actuator’s overall capacities and limitations, control addresses practical implementation and determines usability.

Building on previous research that demonstrates at the simulation level how integrating various soft actuation mechanisms into hybrid systems enhances performance and mitigates individual limitations, the development and testing of this type of system can be achieved using the developed actuation and another soft component, specifically a PneuNet. The control perspective covers both the actuation structure’s controllability and the performance offered by the neural network used as the controller.

### 4.1. Linear Actuator

The primary focus of the actuator design is to determine a strategy for amplifying the displacement performed by the actuator compared to its overall dimensions. SMA materials generally have limited deformations, determined by the specific alloy and shape; the wires found on the market have an average of 4–5% length reduction when activated. The solution developed in the following steps offers a method to encapsulate a considerable wire length in a compact encapsulation along a helical channel. The iterative process is detailed below.

The guidance channel is the first development step and the most essential actuation element. In the initial concept, the driving wire was configured in a sawtooth arrangement in an open space, with motion guided only through screws with a closed ring. The test proved that a significant length could be reduced to a small area using a complete 5 m wire; the measurement guaranteed the concept’s validity but not satisfactory performance.

Based on technical analysis, the coiled shape was deemed suitable for further approaches, as it reduced the size of the actuator due to its compact form. This can provide a significant mechanical advantage and efficiency in converting linear motion into rotational or vice versa.

The first coiled iteration involved a solid tube on which a helical channel with a constant pitch was carved. In the design of helical channels, maintaining a constant pitch results in geometric constraints regarding the linearity of the wire trajectory along the actuator. Specifically, a helical structure with a constant pitch leads to an acute angle formation at the channel’s terminus. This acute angle can disrupt the linear path, thus contradicting design guidelines or even damaging the wire during usage.

The core with constant pitch was fabricated using 3D printing in the first coil iteration. The creation of helical channels presents challenges specifically for SMA wires. Due to the layering technique inherent in 3D printing, SMA wires with thin diameters can potentially become entrapped between the printed layers during actuation. This entrapment restricts the wires’ free movement along the channel, which is critical for optimal performance.

Overcoming these challenges requires advanced manufacturing techniques and materials. Firstly, an industrial lathe enables precise machining and shaping of the core using a semicircular cutting knife, ensuring high accuracy and consistency in the production process. Secondly, creating a variable helix by altering the channel’s pitch along the core helps optimize the component’s mechanical and electrical properties.

**Figure 2:**
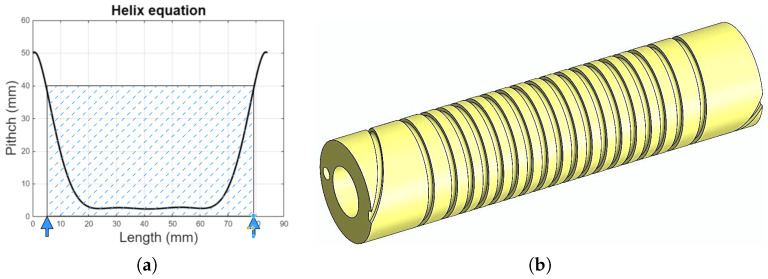
Helical channel development: (**a**) pitch/helix function; (**b**) helical channel on core.

In the present case, the pitch variation was not specified a priori; instead, a polynomial law was empirically identified from the mechanical design (i.e., inferred from the geometry and manufacturing constraints). This polynomial is given in Equation (1) and its profile is illustrated in Figure 2a:

(1) $$Hel\left(l\right)=0.18l^{5}-4.3l^{4}+98.7l^{3}-1060.3l^{2}+1044.3l+50.$$

The channel design depicted in Figure 2b is the solution that emphasizes the control of pitch variation along the coil. This structural modification facilitates a gradual transition to the exterior at the ends, thus minimizing abrupt changes and ensuring a smoother wire trajectory.

During the fabrication process, it was determined that a single coil accommodates approximately 1.2 m of wire. Accounting for a 5% length reduction due to shortening, this results in a 6 cm decrease. Employing two components will result in a twofold increase in expected displacement whilst maintaining the actuator’s overall length and volume within acceptable boundaries.

**Figure 3:**
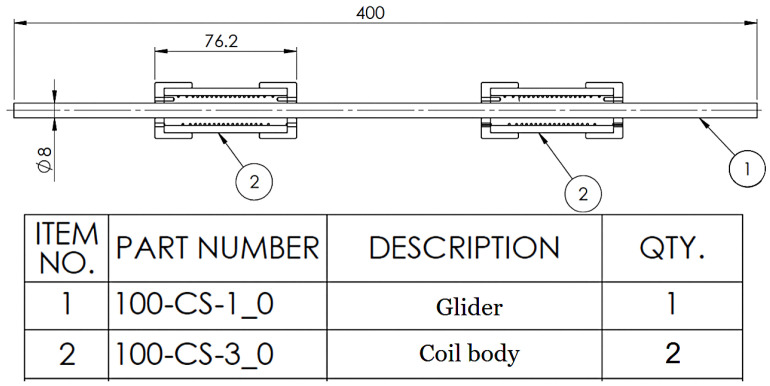
Longitudinal full actuator schematic.

Using a pair of coils requires a solid structure to support the actuator, for which a metallic bar was found suitable. It offers both linear guidance and a stable structure to facilitate core movement with elements arranged as presented in Figure 3. The bar’s metallic properties ensure minimal, flexible deformation, maintaining alignment and enhancing the precision of the actuator’s functionality.

As the fourth principle is fulfilled, the channel’s alignment emerges as a singular point of contention, necessitating rigorous examination. In the initial iteration, the designed supports were affixed to the metallic bar, allowing the capsule to traverse its length unimpeded. It was noted that the wire aligns itself during the actuation. However, the primary issue concerns the protective mechanisms at the ends of the capsule.

**Figure 4:**
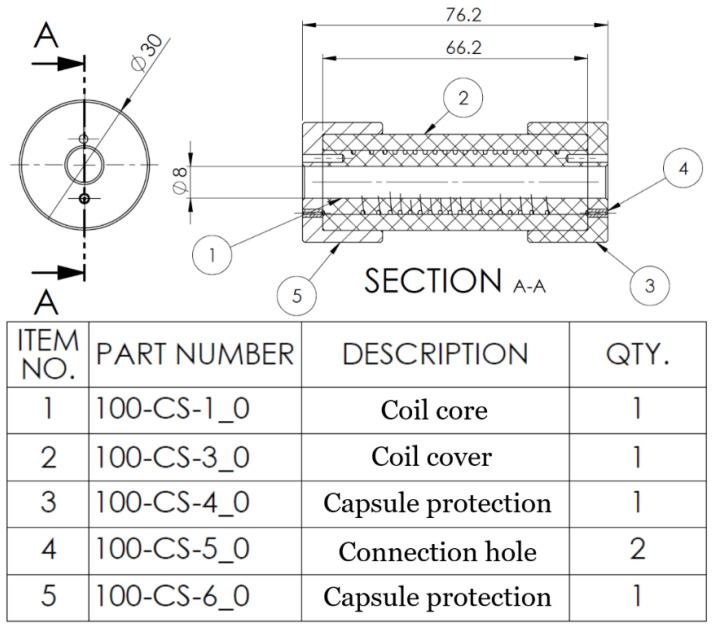
Section of the capsule.

As illustrated in Figure 4, the capsule has two alignment apertures. The aperture near the central axis accommodates a metallic nut that denotes the relative positioning between the core and the protective casing. The other nut is characterized by a diminutive orifice that facilitates a continuum between the external environment and the channel.

The intrinsic elasticity of the wire and the coiled configuration can create an opposite effect, as the wire tends to reshape into its original form: a straight wire. The solution was a simple cover of the coil that creates complete encapsulation for the wire along the channel, observable as element no. 2 in Figure 4.

The employed SMA is a nickel-titanium (Nitinol) shape–memory alloy wire from Dynalloy, Inc. (Irvine, CA, USA) (Flexinol^®^ series) as the contractile element. The wire is a continuous monofilament of nominal diameter 15 μm routed inside a helical grooved channel that constrains the path and promotes repeatable thermo–mechanical cycling. We selected the low-temperature formulation with an austenite–finish temperature of approximately 70 °C, which balances low drive power with safe thermal loads for the surrounding elastomer. A complete specification of material properties and recommended operating limits is provided in the manufacturer’s datasheet [51].

**Figure 5:**
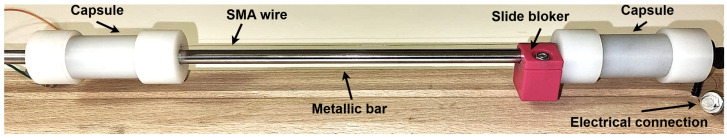
Physical realized actuator.

The fabricated actuator is represented in Figure 5; the SMA wire is barely visible due to its thinness. The actuator is fixed to a wooden plank for use, maintaining a parallel position to the ground.

### 4.2. Control System

The linear actuator is the element that performs the movement, measurable in deformation and force, which represents the driving method for the actuation control system. As a simple linear actuator, the mechanism above only creates directed movement and amplified deformation, offering basic functionality. However, the actuator requires auxiliary elements to form a usable actuation system for practical use, similar to a classical motor.

**Figure 6:**
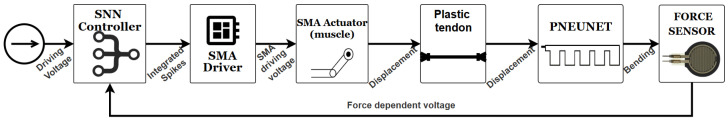
Control loop architecture.

The control loop used in the present work is depicted in Figure 6, representing a particular form of classical feedback control loops. The complete system aims to replicate the movement of a human finger from a neuronal level to external force development and sensing.

The neural network depicts the controller as the primary component in the control loop, apart from the actuator. As previously discussed, a Hardware Spiking Neural Network (HW-SNN) is employed. This network is based on the fundamental characteristics of human neural behavior and functions analogously to a spiking network. This approach aims to enhance the efficiency and robustness of control systems by emulating biological neural processes.

**Figure 7:**
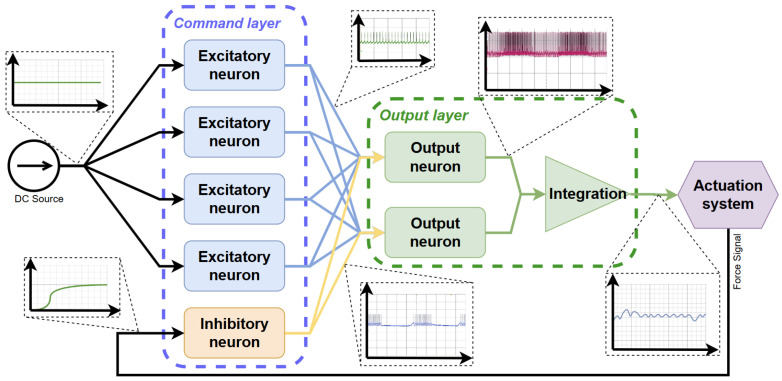
HW-SNN architecture.

Figure 7 illustrates the architecture of the current version of the SNN controller, which incorporates a single feedback signal. The DC signal serves as a step reference that drives the network’s command layer, consisting of four excitatory neurons that generate a combined command signal. Due to the SNN’s non-linear behavior, a direct relationship between the input voltage and the expected displayed force has not yet been established, necessitating a closer examination of the amplitude of the experimental results. This signal is regulated by an inhibitory neuron, which is influenced by a variable signal proportional to the pressing force sensed at the end of the actuation system.

The output layer collects the spiking signal from the command layer, generating an integrated, unified spiking signal to obtain a continuous voltage compatible with the SMA driver. It is noticeable that the effects of excitatory and inhibitory neurons result in a variable final voltage in direct relation to the received force signal.

**Figure 8:**
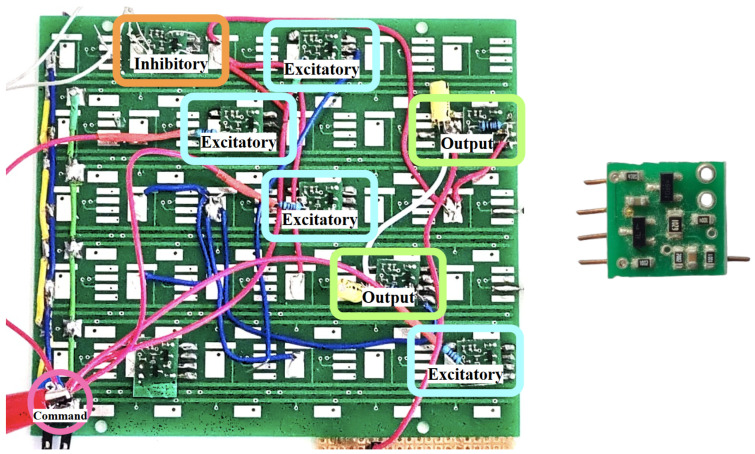
Hardware realization of the controller architecture: network frontplane with labeled inhibitory, excitatory, and output neuron modules (**left**), and a standalone neuron card (**right**) used throughout the prototype.

Following the architecture in Figure 7, we realized the controller as a modular hardware SNN (Figure 8). The backplane hosts identical neuron cards configured as excitatory, inhibitory, and output units; wiring on the board implements the command layer (four excitatory neurons), the feedback path via one inhibitory neuron driven by the FSR, and the output layer that integrates spikes into a continuous command for the SMA driver. This prototype is the physical instantiation of the control loop in Figure 6 and is used for all experiments reported below.

Each neuron card implements the high-plausibility analogue spiking neuron described in [52]. The circuit comprises an electronic SOMA (threshold detection, spike generation, refractory) and an electronic synapse that stores the synaptic weight as a voltage $V_{W}$ on a capacitor $C_{L}$, which can be updated in real time. During activation, a switching transistor generates a spike whose energy/duration scales with $V_{W}$; the postsynaptic potential recovers exponentially with the synapse RC time constant. Potentiation is implemented by two complementary mechanisms: presynaptic post-tetanic potentiation (PTP), modeled by a discharge of $C_{L}$ during activation, and long-term potentiation (LTP), modeled by charge transfer that alters the equilibrium between the auxiliary capacitor $C_{A}$ and $C_{L}$. In the reference design, the supply is low ($V_{DD}\;\approx \;1.6$ V) and the spike event has a short duration (on the order of $t_{\mathrm{SPK}}\;\approx \;44\;\mu s$), enabling fast, low-power operation suitable for embedded control.

The command signal is transmitted to the SMA driver, which powers the actuator as commanded. The driver acts as a voltage-bridging circuit. The actuator’s linear stroke pulls a semi-flexible tendon placed along the actioned part, the PneuNet, similar to a human tendon.

Representing the human finger in terms of flexibility and softness, the PneuNet is driven solely by the SMA actuator via tendon, enhancing the natural muscular features and potential behavior. At the tip of the PneuNet, a force-sensing resistor (FSR) is placed. This resistor generates an analog signal based on the pressure or force applied to its surface, which is transmitted to the SNN through the inhibitory neuron.

The resulting system relies entirely on analog components, providing a rapid response and exhibiting natural behavior from a calculus perspective. Suppose a digital controller, processing unit, or even a software-based neural network were to limit the system’s response speed due to the internal frequency. In that case, this scenario is analyzed in the results Section 5.

### 4.3. Testing Plants

The system evaluation is conducted from a dual perspective: first, assessing the force exerted by the actuator as a driving component within an open-loop configuration; second, performing a comprehensive numerical analysis of closed-loop performance utilizing the SNN as the control architecture. To facilitate the necessary numerical data acquisition, developing two testing environments that adequately represent and capture both open-loop and closed-loop behavioral dynamics is imperative.

Firstly, the open-loop acquisition must cover the range of forces that the standalone actuator performs while driven by different input signals. A clear strategy can be formulated by correlating the SMA current driving from the manufacturer’s technical notes and the testing methodologies found in the literature.

The SMA actuation is conducted via a controlled PWM signal, but with an increased voltage that can produce enough current to heat the metallic alloy. This is achieved via an SMA driver controlled by a PWM signal generated from a digital board.

**Figure 9:**
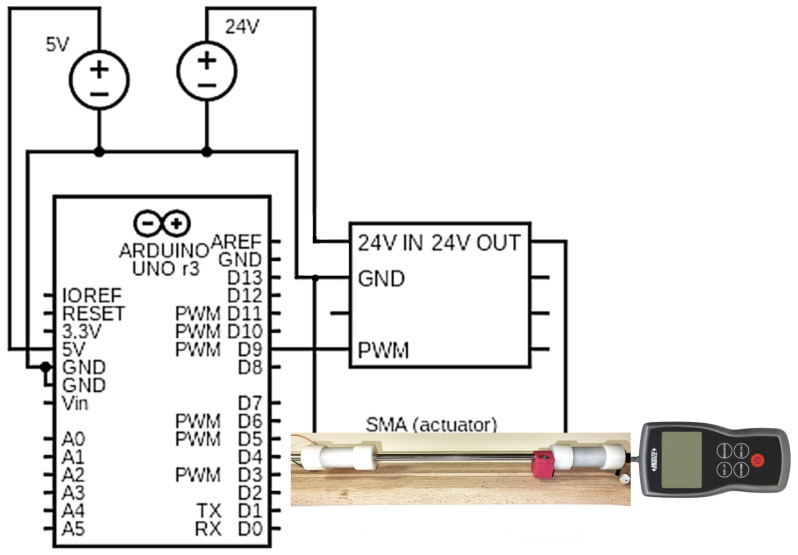
The structure of the microcontroller-based system for actuation of the SMA.

As depicted in Figure 9, the actuation signal is generated by an Arduino Uno Rev3 development board with an ATmega328P microcontroller. The board offers 6 PWM channels, but the maximum voltage it can generate is 5 V, and the maximum current is 50 mA, which does not satisfy the power requirements for the SMA actuation.

The supplementary DC source was added to the plant. It can generate 24 V but is limited to 400 mA current, 410 mA being the recommended maximum current for driving SMA wire. The driver necessary to mimic intermediate electrical actuation is similar to the one developed in parallel work [53], where the electric schema can be found.

Force values are acquired using a force gauge with a precision of 0.005 N and a maximum load of 5 N. The force is measured during an open-loop actuation, during which the force behavior can be observed. Data acquisition is initiated once the force exceeds the minimum detectable threshold of precision.

The driving frequencies represent the minimum, medium, and maximum of the 500 Hz digital PWM output, covering a representative range of dynamics and providing a comprehensive general view of forces. The duty cycle determines the voltage on the wire, as well as the current and temperature produced.

The second analysis focuses on the performance of the closed-loop system, underscoring the capabilities of the HW-SNN in handling a hybrid actuation mechanism. The testing scenario covers a succession of increasing command values, representing step command signals in the permitted voltage interval.

The returned force behaviors are numerically and graphically analyzed and underlie the control system’s performance. The questionnaire is conducted using a similar methodology to the previous experiments, with the Arduino driving board replaced by a neural network that closes the loop with the force sensor.

## 5. Results

### 5.1. Actuator Force Analysis

**Figure 10:**
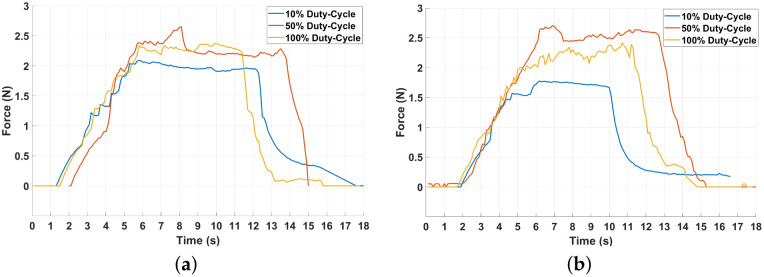
Open-Loop force behavior over 18 s: (**a**) room temperature; (**b**) heated wire.

For the open-loop experiments, the results are displayed in Figure 10, depicting two force evolutions for each duty cycle of the PWM signal. The graphs in the left column present the force starting from the wire at room temperature, and the second experiment begins at the temperature immediately after the first experiment.

Each experiment is run until the pulling force stabilizes at a constant interval, and then the driving signal is stopped to represent the cooling of the wire and the subsequent reduction in force.

A first observation that can be made by examining all behaviors is that the pulling force increases with the rise of the duty cycle. This is a natural behavior, considering the increase in current on the wire, although the relationship is not linear.

As the actuator is designed, the SMA wire slides along a helical channel, directly contacting the coil surface and producing friction. The resulting pulling force is, therefore, reduced, as shown in the graphics. This is a general problem with the current iteration of the actuator and may be approached in a future design.

As depicted in Figure 10, all the exhibited behaviors offer a stable force with almost constant maximum values, making it feasible to use as a driving mechanism. For a deeper understanding, many essential features of the force behavior are numerically collected in Table 1.

The data collected consists of several experiments for each duty cycle. Numerically, a higher duty cycle and, respectively, a higher voltage lead to an increase in all the features of the measurements.

The graphs and the rest of the data also show that for each experiment, the force reaches a stationary value that is not linearly dependent on the PWM. As summarized in Table 1, the average values for each duty cycle studied show that a stationary value is reached with a minimum overshoot. The measured transitory time is close to the rising time, which confirms the non-oscillating behavior. The following factors can describe the key performances:

- Displacement: Within our targeted voltage (5 V) regime, the actuator delivers 41 mm stroke (11.2% of length), i.e., ≈2.2–2.8× the displacement of an equal-length free SMA wire (4–5%).
- Time: From a temporal perspective, the performance is only influenced by the roughness of the metallic bar. It is noticeable that even with this back-hold, the system exhibits fast actuation around 4 s, with a quicker response as the decreasing time increases, showing the SMA’s typical hysteresis.
- Force: The measured friction loss of 0.86 N is non-negligible (≈27%) of the wire’s 3.21 N limit and >30% of the steady-state force. This reflects a first proof-of-concept build with additively manufactured, uncoated polymer channels and non-optimized pre-tension/clearance. Subsequent iterations will target reduced friction via machined or PTFE-lined channels and tuned pre-tension/bend radius (with optional dry-film lubrication).

The benefits brought about by the SMA behavior in the present solution have doubled the exhibited displacement with minimal force losses. The results emphasize the potential of the SMA being incorporated into solutions designed to maintain the principal characteristics. The rules presented and applied during the development stage resulted in a performing actuator that enhanced the capabilities of the SMA wire. The only downgrade is a modest reduction in the exhibited force, due to longitudinal capsule friction and the wire’s curved placement.

### 5.2. Control System Performances

The attached scenario outlines a simple closed-loop performance analysis that begins with the described testing plant. The voltage represents the reference value in the electrical domain. The HW-SNN calculates the error using the inhibitory neuron and realizes the regulator function, transferring the command signal to the SMA driver. The actuator, combined with the plastic tendon and the PneuNet, represents the execution element, with the force sensor acting as a feedback transducer.

**Figure 11:**
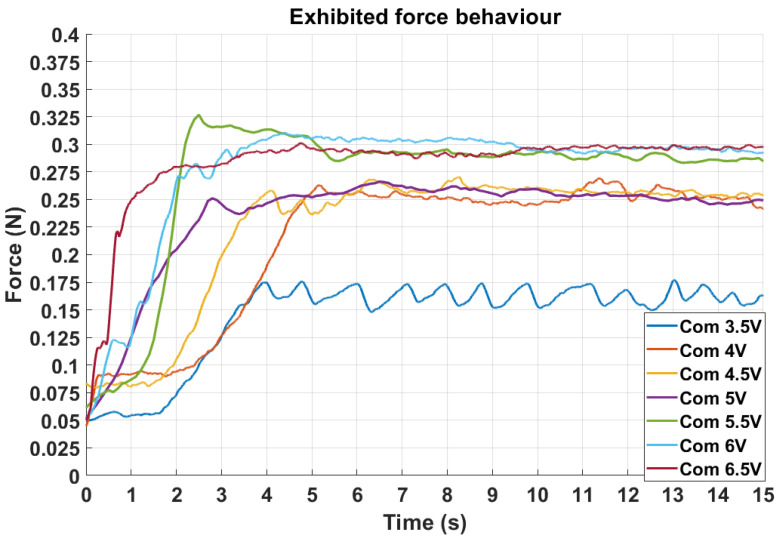
HW-SNN Control loop exhibited behavior.

The analysis focuses on the control system’s produced performance as a complete aggregate and discusses the time and amplitude performances from both graphical and numerical perspectives. The data displayed in Figure 11 presents the force control of the systems, giving an increasing series of reference voltages. The direct relation between the voltages and the force desired is not determined, but a specific general deduction can be made.

As a general observation, the increase in force exhibited with the rise in reference voltage leads to a drivable system structure that can reproduce the changes from input to output. Otherwise, the graphical examples show a reduced variation during the stationary period, suggesting that the system responds to the exhibited force, the network reacting to the produced force.

To assess repeatability and short-term robustness, different testing scenarios were considered several times. Force increases with voltage by about 86% from 3.5 V to 6 V. Variability is lowest around 4–5 V and higher at 3.5 V and 5.5 V (about 0.030 N and 0.059 N). No systematic drifts occurred between repetitions. Overall, 4–5 V is the most stable range, while 5.5 V shows more spread for only a small gain in mean output. Long-term reliability depends on the SMA wire, the pneumatic path and seals, the FSR, and the HW-SNN electronics; endurance testing is planned.

All the experiments exhibited a constant, stationary behavior with slight vibrations within the acceptable limits. The compression force sensed by the sensor is time-dependent. The effect is received by the actuator with a delay significantly smaller than that of an equivalent digital controller.

Table 2 indicates a non-negligible overshoot for the HW-SNN controller (up to 18.47% at 3.5 V). This is consistent with the plant dynamics induced by the SMA thermal inertia and phase-transition hysteresis, which sustain contraction after the input is reduced, the compliance in the tendon/pneumatic path stores and releases elastic energy, and the sensing and control latencies (FSR dynamics and spiking/analog processing) delay the inhibitory response. The effect is accentuated at low commands—near the activation threshold—where nonlinearity and drive quantization are proportionally larger, whereas the 4–5 V band exhibits smaller overshoot. Mitigation includes low-force gain scheduling and an anticipatory clamp of the heating drive as the measured force approaches the setpoint.

The same Arduino board presented in the open-loop tests is used as a digital controller for a comprehensive comparison. In a similar configuration, we used a PWM signal with a duty cycle inversely proportional to the received value. The force-sensing resistor is mounted in a voltage divider, where the measured force is received as a voltage between 0 V and 5 V. The digital controller’s numerical results during this experiment are shown below.

The graphics present only one of the conducted experiments for each reference value, highlighting a general evolution of the system’s behavior across a command interval. A data summary is shown in Table 2 for a detailed numerical perspective.

The numerical data presented in the table represent a representative performance review of all the experiments performed. It is observed that a stronger reference increases the resulting force and time-related performances. In some cases, closer to 5.5 V, results show a more stable behavior offering both speed and accuracy, exceeding the efficiency of the digital controller in some experiments.

**Figure 12:**
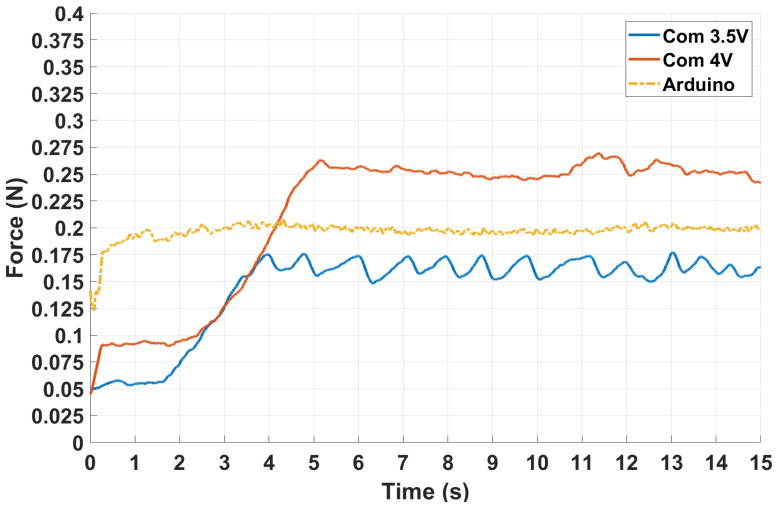
SNN Control versus Arduino digital controller.

Furthermore, the digital controller exhibits a more rapid response in the time domain, primarily attributable to its computational intensity. In contrast, the SNN demonstrates a more gradual and nuanced operational behavior, as shown in Figure 12.

The graphical representations, supported by the corresponding numerical data, indicate that the system’s performance is adequate for practical applications in real-world scenarios. Moreover, employing the SNN as a controller provides functionality that more closely mimics the human neuronal control structure, exhibiting behavior that is more similar to reflexive processes than traditional control architectures.

## 6. Conclusions and Future Work

The present research focuses on analyzing a new linear actuator and its associated control system. To validate the proposed prototype, multiple experiments were conducted. One experiment demonstrates the performance and potential of the newly developed actuator in the context of advancing knowledge in sustainable soft robotics. At the same time, the other experiments illustrate its usability and performance in a control system that closely mimics the behavior of a neural-controlled muscle and finger.

The results of the first experiment reveal a substantial increase in actuator deformation while maintaining a sufficient force–displacement ratio. Specifically, the contraction amplitude reaches nearly 12%, up from the previous 5%, with only a reduction in the actuator’s overall force. Based on the numerical data, the actuator presents great potential as an initial prototype. Considering its reduced overall size and with even more improvement possibilities, it can become a strong alternative to classical linear actuators. The SMA wire length and diameter facilitate this variety of arrangements, which produce amplified motion types and directed movement.

The system reveals high performances, confirming the beneficial combination in hybrid soft actuators, which has been proven both physically and experimentally. One essential feature of the new system is the controllability of the actuator. The HW-SNN exhibits a rapid response and high sensitivity, making it a superior alternative to traditional numerical control structures. The force response evolution demonstrates the effectiveness of the control strategy and the overall system. At the same time, the representation of the human biological neuron-to-finger configuration highlights the system’s potential as a foundational approach for developing biologically accurate prostheses. This representation means a concrete, end-to-end hardware mapping of a minimal sensorimotor loop: an excitatory command population and a feedback-driven inhibitory neuron (controller), a power interface that converts spikes to analog drive, an SMA tendon acting as an artificial muscle, and a fingertip FSR providing negative feedback to the inhibitory neuron (Figure 7 and Figure 8). This preserves the biological organization (local reflex-like inhibition, population command) while remaining low-power and compact. As such, it is a feasible module for embedded prosthetic controllers and a scalable building block for multi-digit humanoid hands, rather than a finished clinical device. Furthermore, this representation lays the groundwork for developing novel humanoid robots.

The research topics that emerge from the proposed design are expected to generate a wide range of applications. Further enhancement of the encapsulation and experimental determination of a more accurate equation for the helical channel or core length can improve the actuator, reducing friction, producing finer movement, and increasing force amplitude. The size of the complete actuator can be reduced by shaping the cumbersome capsule into a pleasing form without adding extra material. Additionally, the inner diameter can be reduced, thereby increasing the embedding capabilities in more complex infrastructures.

Future work includes the development of a new version of the actuator, which is already underway. The main modifications are related to the core channel, length and diameter, helical pitch, and even the number of wires used. Regarding the SMA displacement performance, we acknowledge the necessity of having a thorough analysis on how the chosen wire compares with state-of-the-art soft actuators from the recent literature.

As a small observation, the tendons used in the experiment are unsuitable for prostheses and should be replaced with ones that have bending properties. The control method can also be improved to achieve better performance in everyday use. Future improvements can include scaling the hardware, utilizing more neurons, or implementing parallel configurations to enhance actuation power. The control structure’s versatility enables the creation of dedicated networks to encode multiple sensors, thereby gaining a more comprehensive understanding of the scene.

Following this work, the necessary steps for an optimal design will be considered, while the final version will be submitted for patenting.

## Figures and Tables

**Table 1:** Actuation force numerical summary.

Duty	Rising	Transitory	Overshoot	Stationary	Decrease	Displacement
Cycle	Time	Time		Value	Time	
10%	4.2 s	4.4 s	2%	1.6 N	2.5 s	37.1 mm
50%	4.3 s	4.43 s	≤1%	2.3 N	2.4 s	40.2 mm
100%	3.6 s	4.0 s	≤1%	2.4 N	2.4 s	41 mm

**Table 2:** Performance indices for a range of step commands.

Command	3.5 V	4 V	4.5 V	5 V	5.5 V	6 V	6.5 V	Arduino
RiseTime (s)	3.09	2.73	2.33	2.3	2.22	1.814	1.648	0.272
TransientTime (s)	1.9319	1.72	2.2	1.989	3.1	4.4	1.042	0.424
SettlingTime (s)	1.1277	1.4	1.31	1.47	1.2	1.643	0.907	0.23
SettlingMin (N)	0.05	0.2	0.21	0.212	0.098	0.23	0.274	0.176
SettlingMax (N)	0.177	0.27	0.273	0.278	0.32	0.311	0.311	0.208
Overshoot (%)	18.47	17	9.95	11.57	11.35	18.39	1.795	6.254
Peak (N)	0.177	0.271	0.273	0.278	0.328	0.311	0.311	0.208
PeakTime (s)	13.06	9.36	8.21	6.3	3.1	4.319	13.02	4.315
Mean Force Value (N)	0.15146	0.24373	0.2489	0.24506	0.26326	0.28202	0.29826	0.19791
Stadard deviation (σ)	0.029652	0.0086156	0.0086687	0.010347	0.058728	0.01266	0.0032728	0.0019577

## Data Availability

The data acquired from the physical plant regarding the forces and spiking signal are available at https://github.com/FABrasoveanu/HybridActuator_Data.git (accessed on 1 September 2025).
